# Molecular Players in Hematologic Tumor Cell Trafficking

**DOI:** 10.3389/fimmu.2019.00156

**Published:** 2019-02-06

**Authors:** Javier Redondo-Muñoz, Angeles García-Pardo, Joaquin Teixidó

**Affiliations:** ^1^Department of Immunology, Ophthalmology and ERL, Hospital 12 de Octubre Health Research Institute (imas12), School of Medicine, Complutense University, Madrid, Spain; ^2^Manchester Collaborative Centre for Inflammation Research, Lydia Becker Institute of Immunology and Inflammation, University of Manchester, Manchester, United Kingdom; ^3^Department of Molecular Biomedicine, Centro de Investigaciones Biológicas (CSIC), Madrid, Spain

**Keywords:** hematological cancer, cell trafficking, adhesion molecule, chemokines (CK), immunotherapy

## Abstract

The trafficking of neoplastic cells represents a key process that contributes to progression of hematologic malignancies. Diapedesis of neoplastic cells across endothelium and perivascular cells is facilitated by adhesion molecules and chemokines, which act in concert to tightly regulate directional motility. Intravital microscopy provides spatio-temporal views of neoplastic cell trafficking, and is crucial for testing and developing therapies against hematologic cancers. Multiple myeloma (MM), chronic lymphocytic leukemia (CLL), and acute lymphoblastic leukemia (ALL) are hematologic malignancies characterized by continuous neoplastic cell trafficking during disease progression. A common feature of these neoplasias is the homing and infiltration of blood cancer cells into the bone marrow (BM), which favors growth and survival of the malignant cells. MM cells traffic between different BM niches and egress from BM at late disease stages. Besides the BM, CLL cells commonly home to lymph nodes (LNs) and spleen. Likewise, ALL cells also infiltrate extramedullary organs, such as the central nervous system, spleen, liver, and testicles. The α4β1 integrin and the chemokine receptor CXCR4 are key molecules for MM, ALL, and CLL cell trafficking into and out of the BM. In addition, the chemokine receptor CCR7 controls CLL cell homing to LNs, and CXCR4, CCR7, and CXCR3 contribute to ALL cell migration across endothelia and the blood brain barrier. Some of these receptors are used as diagnostic markers for relapse and survival in ALL patients, and their level of expression allows clinicians to choose the appropriate treatments. In CLL, elevated α4β1 expression is an established adverse prognostic marker, reinforcing its role in the disease expansion. Combining current chemotherapies with inhibitors of malignant cell trafficking could represent a useful therapy against these neoplasias. Moreover, immunotherapy using humanized antibodies, CAR-T cells, or immune check-point inhibitors together with agents targeting the migration of tumor cells could also restrict their survival. In this review, we provide a view of the molecular players that regulate the trafficking of neoplastic cells during development and progression of MM, CLL, and ALL, together with current therapies that target the malignant cells.

## Introduction

The trafficking of hematologic malignant cells follows trajectories that are governed by the functional involvement of adhesion and chemokine receptors expressed by these cells, and their ligands exposed at specific homing sites. The lodging of neoplastic cells at these sites starts with cancer cell diapedesis across endothelia and perivascular cell layers. This is followed by cell migration in response to cell-bound and extracellular matrix (ECM)-bound chemokines as well as to ECM proteins, which guide the tumor cells to permissive niches. Among the various hematologic malignancies, we will focus on three lymphoproliferative disorders: multiple myeloma (MM), chronic lymphocytic leukemia (CLL), and acute lymphocytic leukemia (ALL). A common trafficking step during progression of these malignancies is the neoplastic cell migration into and out of the bone marrow (BM). In addition, CLL cells travel to lymph nodes (LNs), whereas ALL cells infiltrate extramedullary organs such as the central nervous system (CNS). Localization in these niches is beneficial to the malignant cells, as they receive survival and proliferative signals, which contribute to progression of the disease.

MM is the second most common hematologic malignancy and is characterized by the accumulation of malignant plasma cells at multiple sites in the BM. This causes the characteristic multifocal lesions that highlight MM cell ability to traffic into and out of different niches in the BM (1–3). In most cases, MM is preceded by an asymptomatic pre-malignant condition, monoclonal gammopathy of undetermined significance, followed by another asymptomatic phase called smoldering myeloma (1, 2, 4, 5). The latest MM phases are characterized by the egress of MM cells from the BM to the bloodstream, once they become independent from growth and survival signals provided by the BM, a condition named extramedullary disease. MM cells in circulation can subsequently colonize different organs, or develop plasma cell leukemia (6). Alkylating agents, proteasome inhibitors, steroids, autologous stem cell transplantation, and immunomodulatory drugs are the most frequent protocols in MM treatment (7–10). Furthermore, immunotherapy protocols with monoclonal antibodies and CAR-T cells are entering a new era of MM treatment. Yet, although substantial improvement in patient survival has been achieved in recent years, MM remains mostly incurable. In addition, resistance responses to proteasome inhibitors and immunomodulatory agents represent important clinical challenges in MM treatment (7).

CLL, the most common leukemia in Western countries, is characterized by the accumulation of mature CD5^+^ B lymphocytes in the peripheral blood (PB) and the progressive infiltration of lymphoid organs by these cells (11, 12). The traffic of CLL cells between PB and lymphoid organs, as well as the malignant cell retention in these tissues, is regulated by adhesive and migratory molecules and contributes to CLL progression (13, 14). Clinically, CLL is a heterogeneous malignancy, with good or poor prognosis mostly determined by the presence of specific markers, particularly mutated (M-CLL) or unmutated (U-CLL) immunoglobulin heavy-chain variable region (IGVH) (11, 12). Differences in adhesion/migration pathways between M-CLL and U-CLL have also been demonstrated by proteomic analyses, which showed that U-CLL cells have a less migratory and more adhesive protein pattern than M-CLL cells (15). This fact could favor their retention in lymphoid tissues and the presence of lymphadenopathy, as observed in U-CLL patients. Current therapies for CLL include the combination fludarabine-cyclophosphamide-rituximab, as well as the newer compounds ibrutinib (Bruton's tyrosine kinase [BTK] inhibitor), idelalisib (phosphatidylinositol 3-kinase δ [PI3-Kδ] inhibitor), and venetoclax (Bcl-2 inhibitor) (12, 16). Although many patients respond to treatment and some achieve remission, CLL remains an incurable disease.

ALL is the most frequent pediatric cancer and accounts for 20% of adult leukemia (17). ALL leukemic cells can originate from B-cell lymphoblasts (B-ALL, 85% of ALL) or T-cell progenitors (T-ALL, 15% of ALL). T-ALL is relatively rare and characterized by an inferior treatment outcome than B-ALL. Clinically, the standard-risk ALL comprises those patients between 1 and 10 years old (y.o.), hyperdiploidy and the translocation t(12;21) ETV6/RUNX1. In contrast, high-risk includes those patients younger than 1 y.o. or elder than 10 y.o., an initial leukoycte count higher than 50,000 per cubic millimeter, hypodiploidy, and other genomic alterations (18, 19). Currently, standard induction therapy includes several anti-tumor drugs such as prednisone, dexamethasone or vincristine, with or without prophylactic intrathecal therapy. At the end of the induction, the complete remission or the presence of MRD is evaluated, as patients with MRD after chemotherapy present higher risk of relapse and death (18).

ALL cells use similar molecular mechanisms than normal lymphocytes to migrate across physical barriers (20). ALL initiates either in the BM or in the thymus, and leukemic cells may remain in these organs or egress, entering the circulation and infiltrating other tissues such as the spleen, CNS, and testes. ALL cells located in the BM or migrating through other tissues interact with highly complex microenvironments composed of ECM proteins (collagens, fibronectin, laminin, proteoglycans), soluble molecules (cytokines, chemokines, and growth factors), and other cell types (stromal cells, osteoblasts, endothelial cells, and macrophages) (21). Recent evidence, based on the use of an *in vitro* 3D microfluidic system that includes stromal cells, osteoblasts, and B-ALL cells, supports the notion that biophysical properties, such as the matrix stiffness drive ALL progression and dissemination (22).

Integrins are the main adhesion receptors facilitating the trafficking of neoplastic cells. Integrins are heterodimers of α and β subunits that mediate cell-cell and cell-ECM interactions, and connect the ECM with the actin cytoskeleton (23, 24). Additionally, integrin-dependent cell adhesion triggers intracellular signaling that contributes to the control of cell growth and survival (23, 25). Integrins adopt different conformations, which determine their state of activation linked to their ability to bind ligands with high-affinity and to induce subsequent intracellular signaling (26–29). Integrin activation is a dynamic process that can be achieved by several stimuli from outside (outside-in) or inside (inside-out) the cell, a property that highlights the integrin role as main connectors between the cancer cells and their environment (24).

Chemokines are chemotactic cytokines that promote cell migration and activation under homeostatic and inflammatory conditions, and play critical roles during hematopoiesis, immune surveillance and inflammation, morphogenesis, and neovascularization, as well as in the trafficking of hematologic tumor cells (30–32). Chemokines bind to seven transmembrane-spanning receptors coupled to heterotrimeric guanine nucleotide-binding (G) proteins, which transmit intracellular signals for cell adhesion, migration, and survival (30, 33–35). Ligand binding by chemokine receptors involves the receptor N-terminal domain and three extracellular loops, whereas the intracellular loops and the C-terminal region are coupled to receptor internalization and to heterotrimeric G proteins, respectively (35). The conserved DRY motif is located intracellularly, and is critical for coupling the chemokine receptor to G proteins and for transmitting downstream signaling. Several atypical receptors, including CXCR7 and DARC, lack the DRY motif and are unable to associate with G proteins (36) and induce signaling, therefore acting as scavengers for chemokines (37). Besides binding to these receptors, chemokines also interact with glycosaminoglycans (GAGs), and this contributes to chemokine retention on the surface of endothelial cells (38).

Selectins have also been implicated in the initial adhesion steps of the trafficking of hematologic tumor cells. Selectins are a family of C-type lectin receptors divided according to their expression in leukocytes (L-selectin), platelets (P-selectin), or endothelial cells (E- and P-selectins) (39, 40). The roles of these cell surface receptors and their glycosylated ligands have been extensively explored in leukocyte recruitment, granular secretion, and placental development (40, 41). Selectins and their ligands are crucial in multiple physiological and pathological situations, including those related to cancer and immune response (39). Of note, cancer cells present changes in cell-surface glycosylation that are recognized by selectins, galectins, and siglecs (42). For this reason, targeting selectin-ligand interactions has clinical relevance for cancer immunotherapies.

Matrix metalloproteinases (MMPs) are a large family of Zn^2+^-dependent proteases that facilitate cell migration by degrading basement membranes and ECM, as well as by releasing matrix-bound chemokines and growth factors (43). In depth proteomic analyses have demonstrated that MMPs can degrade many other substrates, including cytoskeletal proteins and signaling molecules (44, 45). Additionally, it is now well-established that many MMPs also display non-catalytic activities, which mostly rely on their localization at the cell surface, either *via* their transmembrane domain (MT-MMPs), or by binding to specific cell surface receptors (46). MMP-9 (gelatinase-B) is the most relevant MMP regulating the migration and other functions of lymphocytes.

In this review we summarize the most relevant molecules involved in MM, CLL, and ALL cell trafficking, indicating their function, interconnection, and possible use as therapeutic targets.

## Integrins in Hematologic Tumor Cell Trafficking

### The α4β1 Integrin in MM, CLL, and ALL

Compelling evidence has clearly established that the α4β1 integrin (CD49d/CD29, very late antigen-4, VLA-4) is a key molecule involved in hematopoietic cell trafficking. α4β1 interacts with the IgG domains 1 and 4 of vascular cell adhesion molecule-1 (VCAM-1, CD106) and with the CS-1 site (EILDV sequence) in fibronectin (47). In addition, α4β1 is a receptor for MMP-9 in CLL cells and recognizes the specific sequence VPLDTHDVFQ, located in blade 4 of the MMP-9 hemopexin domain (48, 49). Besides contributing to lymphocyte trafficking to sites of injury and infection, α4β1 plays key roles during lymphopoiesis and myelopoiesis in the BM (50).

**Figure 1:**
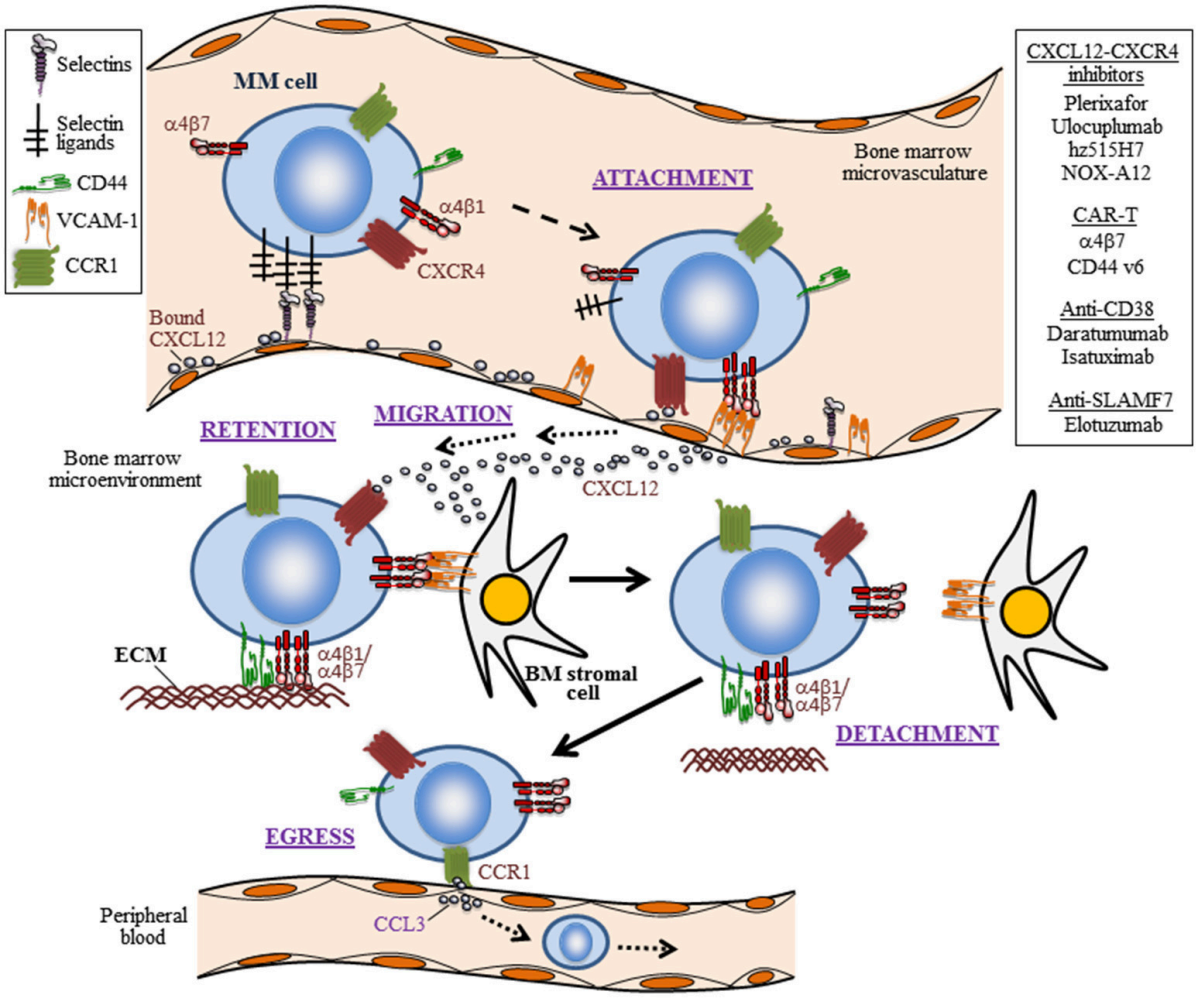
The trafficking life of MM cells. Depicted are five steps (attachment, migration, retention, detachment, and egress) in the trafficking of myeloma cells into the BM and their escape to the periphery, as well as some of the involved trafficking receptors and their ligands. The initial attachment of MM cells to the BM microvasculature is controlled by selectins, the CXCL12-CXCR4 axis, and the α4β1 integrin interaction with VCAM-1. Importantly, CXCL12-CXCR4 leads to upregulation of α4β1-dependent MM cell adhesion. The migration and retention of MM cells in the BM is contributed by the CXCL12-CXCR4 chemoattraction module, by adhesion mediated by the integrins α4β1 and α4β7, as well as by CD44. Ligands for these adhesion receptors are components of the extracellular matrix (ECM), such as fibronectin and hialuronic acid. Weakening or disrupting these adhesive interactions causes MM cell detachment from the BM microenvironment and egress to peripheral blood. The homing of MM cells to the BM can be inhibited by the CXCL12 inhibitor NOX-A12, and neutralizing CXCL12 binding to CXCR4 with the plerixafor blocks MM cell interaction with the BM microenvironment. A putative active cell egress mechanism is proposed as depicted by the CCL3-CCR1 interaction. Not shown is the trafficking of circulating MM cells to extramedullary colonization sites. In addition to therapies targeting the CXCL12-CXCR4 axis, the use of CAR T cells addressing α4β7 and CD44, and humanized monoclonal antibodies against CD38 and SLAMF7, which are currently being tested in MM, are shown.

**Figure 2:**
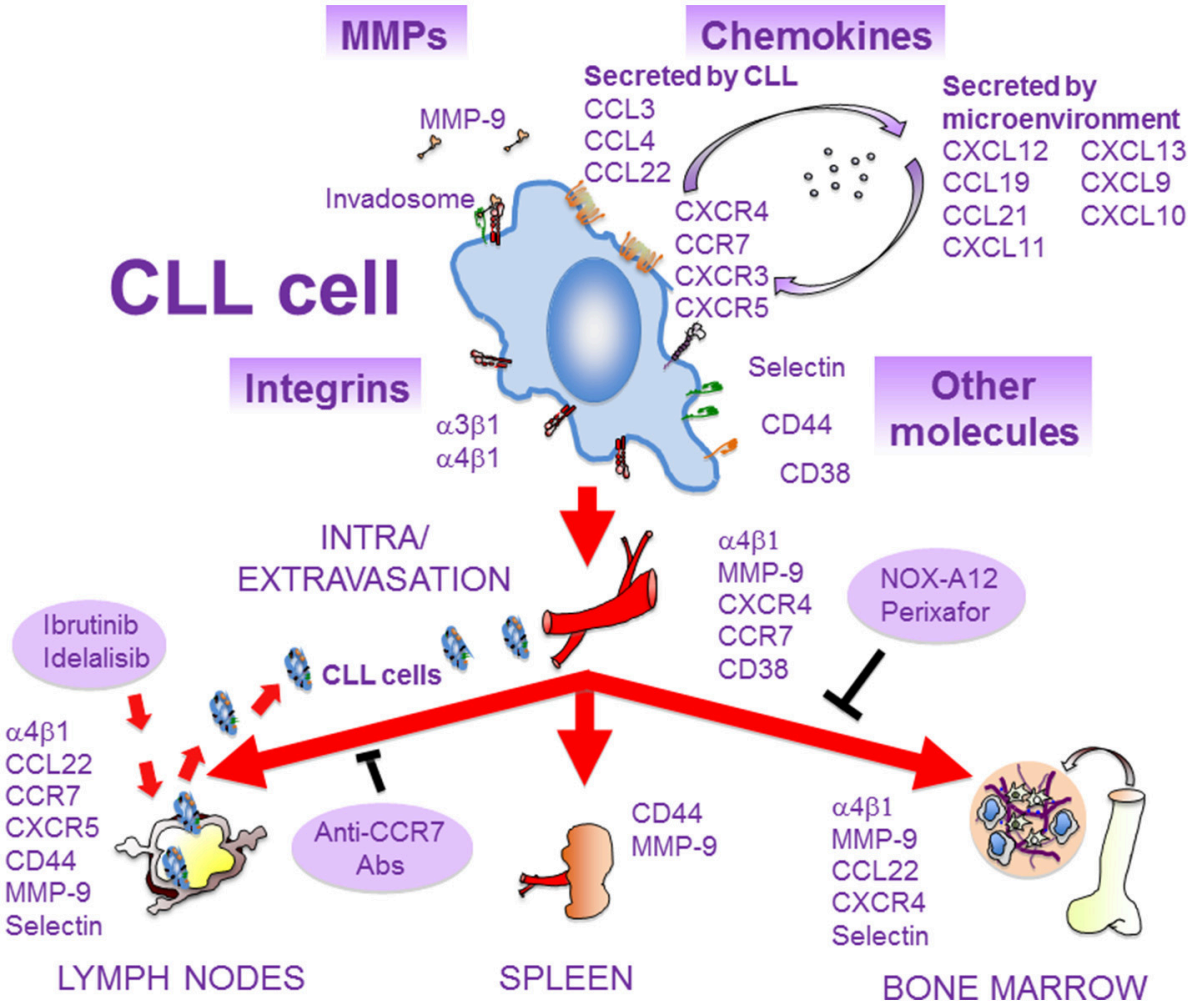
Molecules involved in the migration of CLL cells. As CLL progresses, malignant cells extravasate and infiltrate the BM and secondary lymphoid organs. This process is mediated by several molecules, expressed and/or secreted by CLL cells or by the microenvironment of lymphoid tissues. Some of these molecules interact physically and/or functionally and may constitute the CLL cell invadosome. The α4β1 integrin is critical for CLL cell homing to BM and LNs, and blocking BCR signaling with ibrutinib or idelalisib inhibits α4β1 function and results in lymphocytosis. The chemokine receptors CCR7 and CXCR4 are crucial for CLL cell traffic to LNs and BM, respectively. These receptors can be blocked by specific antibodies or inhibitors (NOX-A12, perixafor). MMP-9, CD44, and CD38 are also important for CLL cell migration and localization in lymphoid niches. Adhesive interactions with stromal cells in these niches, mediated by some of the depicted molecules, favor survival and chemoresistance of CLL cells and contribute to disease progression.

**Figure 3:**
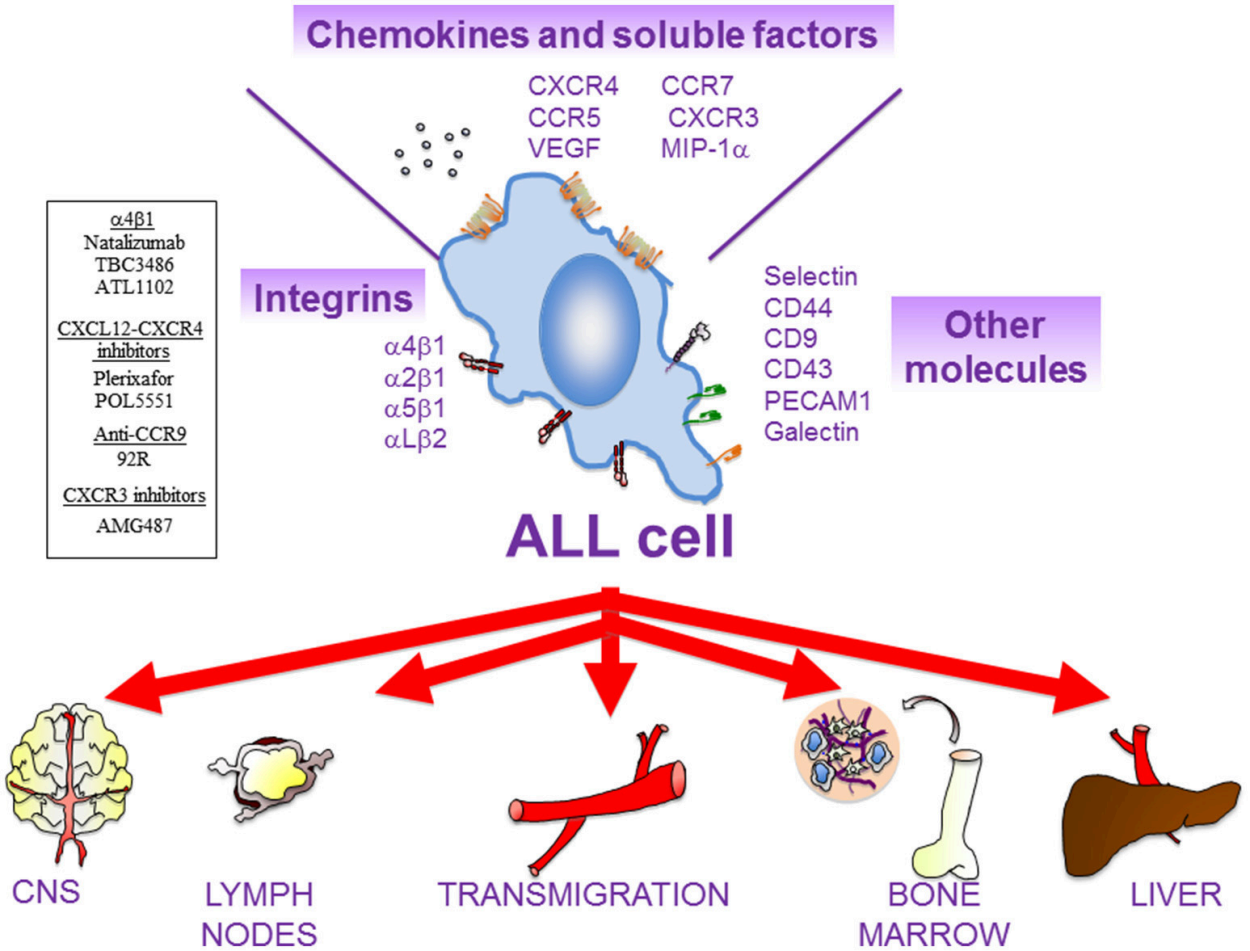
Main actors of ALL cell migration. There are multiple cell surface receptors and soluble molecules that drive ALL cell movement. ALL cells might remain in the BM or colonize multiple extramedullary organs, such as central nervous system (CNS), lymph nodes (LNs), liver, and testes. In general, β1 integrins (α4β1, α2β1, α5β1) and the integrin αLβ2 are critical for the homing of ALL cells into the BM and for their infiltration in most of the extramedullary organs. The chemokine receptor CXCR4 mediates ALL cell infiltration into BM, liver, lung, and CNS, whilst CCR7 controls ALL infiltration into CNS and LNs. CXCR3 is also critical for CNS infiltration. Other molecules that might contribute to ALL migration includes MIP-1α, VEGF, PECAM-1, VE-Cadherin (for CNS infiltration); IL-7 (for LNs); and CD44 (for BM). Several compounds targeting molecular players in ALL cell migration that are currently being tested in preclinical trials are shown on the left.

The attachment of MM cells to the α4β1 ligands VCAM-1 and fibronectin, which are present in the BM microenvironment (Figure 1), was recognized early (51, 52) and later shown to contribute to MM progression in *in vivo* models (53, 54). We recently demonstrated by intravital imaging a key role of α4β1 in MM and CLL cell attachment to the BM microvasculature (55) (Figures 1, 2). Furthermore, *in vivo* experiments have demonstrated that blocking α4β1 function with specific antibodies abolishes homing of CLL (55–57) and primary B-ALL cells (58–60) to BM and LNs (Figures 2, 3). The α4β1 integrin is expressed in ~40% of CLL cases, and α4^+^ cells show increased migratory capacity when tested *in vitro* (61). Indeed, BM infiltration by CLL cells directly correlates with the levels of α4β1 expression (57, 62), and high α4β1 levels correlate with early development of lymphadenopathy (56, 63, 64). Likewise, minimal residual disease tumor cells from myeloma BM samples have high α4β1 expression (65), whereas the levels of this integrin are much lower in circulating MM cells (66). This evidence reveals that α4β1 plays a crucial functional role in the engraftment and progression of these hematologic malignancies.

Because CLL expansion relies on the ability to home and locate in lymphoid tissues, α4β1 constitutes a robust, independent prognostic marker, and its high expression (>30% positive cells) adversely correlates with overall and progression-free survival (67–69). In line with this, α4β1 was shown to be overexpressed in CLL cases with trisomy 12, a genetic alteration associated with higher cell proliferation and disease progression (70). Additionally, *in vitro* and *in vivo* phenotypic studies have shown that transendothelial migration regulates α4β1 expression, as LN-derived CLL cells have higher α4β1 levels than PB-CLL cells (71, 72). α4β1 is also regulated by the B-cell receptor (BCR), and targeting this receptor constitutes a therapeutic option in CLL (see below). α4β1 is also an independent risk factor in pediatric B-ALL, where high α4β1 expression associates with worse probabilities of relapse-free and overall survival (58, 73). Interestingly, low α4β1 levels correlate with adverse survival in a cohort of adult patients with T- and B-ALL (74), suggesting that α4 expression associates with poor outcome only in pediatric patients.

Besides its key role in cell migration, the interaction of α4β1 with its ligands favors proliferation, survival, and chemoresistance in hematologic malignancies. For example, the α4β1-dependent CLL cell adhesion regulates proteins of the Bcl-2 family and induces cell survival signaling (75–79). Cell adhesion-mediated drug resistance (CAM-DR) is a process involved in chemoresistance and operates in B-ALL (80) and in the response of MM cells to the proteasome inhibitor bortezomib (BTZ) (81–83). It was also demonstrated that MM cell attachment to BM stroma involving α4β1 activates the MAP kinase and NF-κB pathways, increases cell cycle regulatory and anti-apoptotic proteins and induces IL-6 secretion, overall stimulating MM cell growth, survival, and migration (1). In other studies, β1 integrins, likely α4β1, were shown to cooperate with IL-6 to induce STAT3 signaling and Pyk2 phosphorylation (84, 85), and to contribute to MM cell survival. The α4β1/VCAM-1 interactions also contribute to the defective production of normal blood cells and to the tumor-associated osteolysis in the BM of aggressive B-ALL (86). The crucial role of α4β1 in MM, CLL and ALL cell trafficking, proliferation, survival, and chemoresistance is schematically depicted in Figures 1–3.

### Other β1 Integrins in Hematologic Malignancies

Leukemic cells also express other members of the β1 integrin subfamily. The α3β1 integrin is widely expressed in CLL cells, and its combined expression with L-selectin (CD62L) and ICAM-1 (CD54) constitutes a good prognostic marker for the disease (87). The only function described so far for α3β1 in CLL is the ability to mediate cell migration on laminin-332, a protein present in LNs (88). The consistent expression of α3β1 integrin in CLL cells is intriguing and deserves further studies. The α2β1 integrin was shown to regulate adhesion and pro-survival signals of T-ALL cells (89). Additionally, the α5β1 integrin is highly expressed in Ph^+^ (Philadelphia chromosome positive) B-ALL cells and controls their adhesion and engraftment in xenograft mice models (90). These β1 integrins may therefore be considered potential targets to control leukemic cell dissemination and organ infiltration.

### The β7 Integrins in MM

Another integrin expressed on MM cells and which shares with α4β1 the α4 subunit is the lymphocyte homing receptor α4β7 (91) (Figure 1). The α4β7 integrin interacts with mucosal vascular addressin cell adhesion molecule 1 (MAdCAM-1) and with fibronectin (92–94), both expressed in the BM microenvironment (95). Similar to α4β1, the activity of α4β7 can be regulated by chemokines, including CXCL12 (96). Recent data indicate that α4β7 might constitute a useful target for chimeric antigen receptor (CAR) T cells in MM (91) (see below). The β7 subunit can also associate with the αE subunit to form the αEβ7 integrin, which mediates cell adhesion to E-cadherin (97, 98). Since BM stromal cells express E-cadherin (99), MM cells use both αEβ7 and α4β7 integrins to attach to BM stroma (100, 101), and these interactions contribute to MM cell lodging in the BM (100). The expression of β7 integrins on MM cells is driven by the oncogene c-maf (101), and correlates with poor survival outcome (100). Interestingly, hypoxia in MM leads to decreased expression of E-cadherin, thus reducing MM cell attachment to BM stroma and enhancing egress of these cells to the circulation (102).

### The αLβ2 Integrin in CLL and ALL

The αLβ2 integrin (CD11a/CD18, leukocyte function-associated antigen-1, LFA-1) is a key adhesion receptor for leukocyte transendothelial migration into lymphoid tissues and sites of inflammation, and interacts with its ligands intercellular cell adhesion molecule (ICAM)-1, -2, or -3 (103). αLβ2 is expressed in many CLL cases, but its function is not fully understood. Compared to normal B cells, several groups have demonstrated defective signaling mechanisms in response to chemokines that leads to deficient αLβ2 activation in CLL (104, 105). The impaired CLL cell motility and transendothelial migration could be overcome by further stimulation of αLβ2 as well as α4β1 with VEGF (104). αLβ2 does not seem to be required for *in vivo* homing of CLL cells to LN or BM, or for CLL cell adhesion to stromal cells, at difference with α4β1 (63, 106). Further studies are needed to clarify the role of αLβ2 in CLL and its contribution to CLL pathology.

In contrast to CLL, the blockade of αLβ2 was shown to affect T-ALL cell adhesion and survival on BM stromal cells (107). Additionally, B-ALL cells with high αLβ2 levels are able to promote leukemia and CNS infiltration in mouse xenograft models (108). On the other hand, CD7 was shown to upregulate the β2 integrin subunit in the B-ALL cell line Tanoue, impacting in the adhesion and extramedullary invasiveness of these cells (109). The apparently different role of αLβ2 in ALL and CLL may suggest that distinct molecular mechanisms or kinetics orchestrate cell migration in these malignancies.

## Chemokines and Their Receptors in Hematologic Tumor Cell Trafficking

### The CXCL12-CXCR4 Axis in Hematologic Malignancies

CXCR4 is a main chemokine receptor expressed by MM, CLL and ALL cells (Figures 1–3). Interaction of CXCR4 with its ligand CXCL12 plays a critical role in the trafficking of these neoplastic cells to the BM (13, 110, 111). The CXCL12 chemokine is highly expressed in active MM by BM stromal and endothelial cells, as well as by MM cells (112–114), and its expression is associated with BM areas with high MM cell infiltration (115). CXCL12 expression can be regulated by TGF-β1 and HIF-2α, with important functional adhesive and migratory consequences (116, 117). Previous studies using the myeloma 5TMM mouse model demonstrated the involvement of the CXCL12/CXCR4 axis in the homing and progression of MM (118), and revealed the reduction of both processes by *in vivo* neutralization of CXCL12 with olaptesed-pegol (NOX-A12) (115). Furthermore, blocking CXCL12 binding to CXCR4 with the plerixafor (AMD3100) inhibitor disrupts MM cell interaction with the BM microenvironment (119), causing MM cell mobilization into the circulation (120) (Figure 1). Plerixafor was also shown to prevent B- and T-ALL cell transendothelial migration and homing into the BM (Figure 2), as well as their extramedullary infiltration in liver, lung and CNS (121–123), highlighting the key role of the CXCL12-CXCR4 module in malignant cell trafficking.

The expression of CXCR4 varies during the course of MM, CLL, and ALL. It was recently reported that MRD subclones in MM express high levels of CXCR4 (65). Increased CXCR4 amounts correlates with the acquisition of an epithelial-mesenchymal transition phenotype, favoring MM cell invasion and bone metastasis (124). In CLL cells, CXCL12 binding induces CXCR4 endocytosis, thus reducing the surface expression of this receptor (125). This fact serves to distinguish peripheral blood CXCR4^high^ CLL cells from CLL cells derived from lymphoid tissues, which are CXCR4^low^. CXCR4 is the most abundant chemokine receptor expressed by B- and T-ALL cells (126), and its elevated expression has been extensively correlated with poor prognosis in ALL patients (86).

The mechanisms that regulate the expression of CXCR4 in hematologic malignancies have therefore been the focus of intense investigations. These studies have shown that hypoxia in the BM leads to increased CXCR4 expression in MM cells, resulting in enhanced migration and homing of circulating MM cells to new BM niches (102, 127). CXCR4 expression can also be stimulated by Notch signaling, and blockade of this signaling leads to reduced MM cell infiltration in the BM (128). On the other hand, using a mouse MM model, it has been reported that treatment with BTZ reduces CXCR4 expression, which might favor MM cell egress from the BM milieu and promote extramedullary disease (129). In CLL, CXCR4 expression is regulated by BTK and its downstream target PIM, and both kinases phosphorylate CXCR4 at Ser 339 (130, 131). Using the Eμ-TCL1 murine model of CLL (132), as well as human CLL cells, Chen et al. (130) showed that BTK inhibition by ibrutinib decreased CXCR4 membrane expression along with a rapid release of CLL cells from spleen and LNs to the circulation. In addition, inhibition of PIM by the small molecule SEL24-B489 also blocked CLL cell migration by reducing CXCR4 surface expression and CXCR4-dependent mTOR activation (131). For acute leukemias, it has been reported that calcineurin signaling promotes the expression of cortactin in T-ALL cells, which in turn controls the levels of CXCR4 at the surface of these tumor cells (133, 134).

CXCR4 can also be regulated by tetraspanins, a family of proteins with four transmembrane domains that play important roles in molecular trafficking and in cell adhesion mediated by integrins (135). For instance, the tetraspanin CD63 interacts with CXCR4 on activated B cells and downregulates this receptor (136). Using *in vivo* models and the B-ALL cell lines REH and Nalm-6, the tetraspanin CD9 was shown to modulate CXCR4-mediated cell migration involving Rac1 signaling, as well as tumor cell survival and homing into the BM and testes (137).

CXCL12 signaling *via* CXCR4 is critical for the regulation of hematologic tumor cell adhesion. The tight functional links in signaling between α4β1 and CXCR4 in MM were revealed by the demonstration that CXCL12 rapidly and transiently stimulates α4β1-dependent MM cell adhesion (138), involving RhoA and Rac1 activities (139, 140). In addition, talin and kindlin-3 positively regulate CXCL12-stimulated, α4β1-dependent MM cell attachment, whereas ICAP-1 (Integrin Cytoplasmic domain-Associated Protein-1) negatively controls this adhesion (55). Moreover, sphingosine-1-phosphate (S1P) stimulates CXCL12-promoted MM cell adhesion to α4β1 ligands (141), and targeting S1P with FTY720 reduces CXCR4 cell-surface levels and inhibits *in vitro* and *in vivo* MM cell migration toward CXCL12 (142). Spatially, CXCL12-dependent α4β1 activation has been shown to directly correlate with restricted lateral diffusion and integrin immobilization in T cells (143), and hence it might also represent a mechanism for spatial regulation of α4β1 by CXCL12 in MM cells. Therefore, the functional links between the CXCL12-CXCR4 axis and the α4β1-mediated cell adhesion provide an essential contribution to blood cancer cell homing to and retention in the BM.

In addition, *via* promoting cell adhesion and migration, the CXCL12-CXCR4 interaction also stimulates cell survival. Thus, besides decreasing CXCR4 expression and altering CLL cell trafficking, ibrutinib, and SEL24-B489 also inhibited CXCL12/CXCR4-mediated CLL cell-tumor microenvironment cross-talk signaling, such as induction of cell survival and CD20 upregulation (131, 144). Similarly, the CXCL12-CXCR4 axis was shown to be critical for leukemia-initiating cell activity and disease progression in primary xenografts of T-ALL cells (133, 134).

CXCR7, a chemokine receptor that may function as a non-signaling scavenger for CXCL12, is expressed in active MM, and inhibition of both CXCR4 and CXCR7 with plerixafor and NOX-A12 functionally interfered with MM cell chemotaxis to the BM, and re-sensitized these cells to proteasome inhibitors (145). Similar to MM, CXCR7 is highly expressed in T-ALL cells compared to normal lymphocytes, and contributes to CXCL12-mediated cell migration (146). Additional *in vivo* studies using relevant mouse models are needed to assess the functional implications and relevance of CXCR7 in MM and T-ALL.

Macrophage migration inhibitory factor (MIF) is a CD74 ligand that can also bind CXCR4 and CXCR7 (147). Interestingly, high MIF levels were detected in MM bone marrow in association with poor survival, and MIF silencing downregulated MM cell adhesion to BM stroma and led to extramedullary spread of the MM cells in SCID mice (148). The implication of MIF in MM progression deserves further studies.

### Role of CCL3, CCL4, CCL22, and CXCL8 in Hematologic Tumor Cell Trafficking

CLL cells secrete several chemokines upon stimulation, mainly CCL3, CCL4, CCL22, and IL-8 (13, 149) (Figure 2). CCL3 and CCL4 are upregulated and secreted after BCR stimulation or when co-cultured with nurse-like cells, indicating that interactions of CLL cells with the microenvironment increase the expression of these chemokines (13, 149). Because CLL-derived monocyte-macrophages, as well as T cells, express CCR1 and CCR5, the receptors for CCL3 and CCL4, respectively, their secretion by CLL cells may serve to attract macrophages and other cells to CLL niches. Therefore, CCL3 and CCL4 may play a role in the regulation of CLL cell interactions with other cells in lymphoid tissues. These interactions contribute to CLL cell survival, and a recent study has demonstrated that macrophages induce survival signals in CLL *via* CCR1-dependent upregulation of the anti-apoptotic protein Mcl-1 (150). Consistent with the above observations, the plasma levels of CCL3/CCL4 are elevated in CLL patients with adverse prognosis. Additionally, Zucchetto et al. (151) showed that CCL3 and CCL4 are overexpressed in CD49d^+^/CD38^+^ CLL cells (poor prognosis), compared to CD49d^−^/CD38^−^ CLL cells, and that ligand engagement of CD38 upregulated both chemokines in the double positive cells.

CCR1 is also expressed in MM cells (152, 153). High CCR1 expression confers poor prognosis in newly diagnosed MM patients, and is associated with increased circulating MM cells (127). Interestingly, CCL3 abrogated MM cell migration toward CXCL12, raising the possibility that the CCL3/CCR1 axis might actively promote MM cell egress from the BM, perhaps competing with retention signals from the CXCR4-α4β1 axis (Figure 1). This active migration from the BM could be similar to the alterations in chemokine receptor expression in lymphocytes egressing from the LNs (154).

Ghia et al. (155) reported an attracting role for the chemokine CCL22 in CLL. They showed that CLL cells from LNs or BM, but not from PB, constitutively express and secrete CCL22. Upon CD40 ligation, PB CLL cells also expressed and secreted CCL22 to the culture media. Indeed, these media induced the migration of FoxP3^+^ regulatory T cells, characterized by high expression of CCR4, the receptor for CCL22. Interaction of CLL cells with Treg in lymphoid tissues through CD40-CD40L may provide survival signals to CLL cells, such as the upregulation of anti-apoptotic proteins (156).

CLL cells stimulated *via* CD40 or CD74 were also shown to secrete CXCL8 (IL-8) (157), and CXCL8 plasma levels correlated with CLL survival, suggesting a possible prognostic value for this chemokine (158). However, a recent study (159) demonstrated that highly purified CLL cells do not produce CXCL8, either constitutively and upon activation, do not express the CXCL8 receptors CXCR1 or CXCR2, and therefore, do not respond to this chemokine. Instead, CXCL8 was released by a small amount of contaminating monocytes present in the culture. The role of CXCL8 in CLL therefore needs to be re-evaluated. In the case of acute leukemias, it has been reported that CXCL8 enhances B-ALL cell adhesion to BM mesenchymal stem cells (160).

### CCR7, CXCR3, and CXCR5 in Hematologic Tumor Cell Trafficking

CCR7 is the receptor for CCL19 and CCL21, two chemokines expressed by high endothelial venules or within lymph nodes that drive lymphocyte homing to LNs (56, 149) (Figure 2). Like in the case of the α4β1 integrin, CCR7 overexpression in CLL cells correlates with the presence of lymphadenopathy, and an anti-CCR7 monoclonal antibody inhibits *in vitro* CLL cell migration and induces complement-dependent cytotoxicity against CLL cells (56, 161, 162). The same antibody also drastically reduced tumor burden and dissemination in xenograft models of human mantle cell lymphoma (163), further supporting its potential therapeutic use in both malignancies. Additionally, we have shown that the CCL21/CCR7 axis upregulates MMP-9 involving ERK1/2 activation, thus suggesting a role for MMP-9 in LN infiltration by CLL cells (164) (Figure 2). The expression of the CCR7 chemokine receptor is higher in T- than B-ALL cells, which strongly correlates with CNS infiltration (165). Furthermore, CCR7 is also critical for the infiltration of T-ALL cells into LNs (166).

CXCR3 is the receptor for the CXCL9, CXCL10, and CXCL11 chemokines, which are present in the serum of CLL patients, with higher levels in U-CLL compared to M-CLL cases (167). The cell surface expression of CXCR3 is variable in CLL, and low expression correlates with advanced disease and other prognosis markers (149, 168). In a more recent study, CXCR3 levels were also shown to inversely correlate with the activation status of CLL cells, that is, with their proliferative capacity (167). These authors showed that the combined expression of CXCR3 and CXCR4 has prognostic value in CLL, and that the CXCR3^low^/CXCR4^high^ pattern correlated with shorter time to the first treatment. They also demonstrated that CXCR3 engagement specifically diminished both CXCR4/CXCL12-directed chemotaxis and α4β1 integrin-mediated cell tethering, thus having a negative regulatory role on the activity of these migration and adhesion receptors. Additional detailed mechanistic studies should help establish the significance of CXCR3 in CLL progression.

The CXCR5 chemokine receptor regulates CLL cell homing to LNs and its main function is the positioning of B cells within lymphoid follicles (169). CXCR5 is the receptor for CXCL13, a chemokine constitutively secreted by stromal cells in these follicles (170). CXCL13 binding to CLL cells induces CXCR5 endocytosis, actin polymerization and activation of ERK1/2 kinases (170). CXCR5 is overexpressed in CLL cells, particularly in cases with nodal involvement, but its expression levels are similar to those in normal CD5^+^ B cells (170, 171). The crucial role for CXCR5 in CLL cell migration and expansion was demonstrated using the Eμ-TCL1 mouse model and intravital imaging (172). The authors showed that CXCR5 guided leukemic cells to splenic B-cell follicles, where they interacted with resident dendritic cells favoring proliferation and disease progression. Additionally, CLL cells stimulated stromal cells *via* lymphotoxin-β-receptor activation, leading to CXCL13 release. Moreover, targeting CXCL13/CXCR5 and lymphotoxin-β-receptor signaling abrogated the proliferative and survival advantage of CLL cells in these niches and retarded disease progression.

## Matrix Metalloproteinases

MMP-9 is the main MMP expressed by CLL cells (173–175), and elevated MMP-9 intracellular levels correlate with advanced disease and poor patient survival (174). Correlation of MMP-9 as well as MMP-14 levels with survival, but not with peripheral organ infiltration, was also observed in relapsed pediatric patients with B- and T-ALL (176). In contrast, MMP-2 expression correlates with an invasive B-ALL phenotype in adult but not in pediatric patients (177).

Although MMP-9 is a secreted protein found in the serum of CLL patients and in CLL cell culture supernatants, it is also consistently detected at the CLL cell surface (48, 174). This localization involves binding to a docking complex formed by α4β1 integrin and 190 kDa CD44v (48) (Figure 2), and may serve to concentrate the MMP-9 catalytic activity at the cell periphery and facilitate cell migration and invasion. However, localization of MMP-9 to the cell membrane also has other important consequences that contribute to CLL pathology. For example, we have demonstrated that MMP-9 binding to α4β1 in CLL cells induces a Lyn/STAT3/Mcl-1 signaling survival pathway, and this function does not require the MMP-9 catalytic activity (78). *In vitro* and *in vivo* experiments have also shown that CLL cell migration requires optimal MMP-9 expression, and that above these optimal levels migration is inhibited (178, 179). This effect is partly due to the downregulation of migration regulatory pathways, such as those involving the GTPase RhoA, Akt, ERK, and FAK, as well as to the upregulation of p190RhoGAP and PTEN, and also implicates catalytic and non-catalytic MMP-9 activities (178, 179). Whether MMP-9 also regulates other molecules with migratory functions in CLL deserves further studies. Importantly, the dual regulatory role of MMP-9 operates *in vivo*, since contact with stroma increases cell-bound MMP-9, and CLL cells from lymphoid tissues express more MMP-9 than their PB counterparts. Elevation of cell-bound MMP-9 at these sites may help the retention of malignant cells in tissues, therefore contributing to disease progression.

## CD44 and CD38

CD44 was originally defined as a homing receptor and its role in the expansion of many solid tumors has been widely documented (180). The involvement of CD44 variants in the *in vivo* MM cell homing to the BM was early shown using the 5T33MM mouse model (181, 182). CD44 may play an important role in the retention of MM cells in the BM (Figure 1), since MRD subclones in the BM of MM patients express high levels of CD44 (65). The potential contribution of CD44 in MM cell trafficking was also reported for MM extramedullary disease, as samples from liver and pleural effusions displayed high CD44 expression (183, 184), indicating that CD44 likely facilitates MM cell attachment at multiple steps of the MM cell trafficking life.

Elevated levels of soluble CD44 are found in the serum of many CLL patients, in correlation with advanced disease (185). CLL cells express CD44s and CD44v and their expression increases upon cell activation (185). Using murine models and human samples, a major role for CD44, particularly CD44v6, in CLL cell homing, engraftment and proliferation in the spleen has been demonstrated (185). CD44v6 is also involved in ALL cell infiltration and altered BM localization (186) (Figure 3). As a functional co-receptor for MMP-9 (48, 187), CD44v likely contributes to CLL cell retention in lymphoid organs, where MMP-9 concentration is high. CD44 may thus function as a migration stop-signal in CLL (Figure 2). In agreement with this, activation of CLL cells by T cells in lymphoid organs induced high avidity CD44-hyaluronan interactions, mostly involving CD44v6, which impaired migration and induced CLL cell arrest on immobilized hyaluronic acid (185).

As in the case of the α4β1 integrin, high expression of CD38 (>30% cells) constitutes a marker for poor prognosis in CLL (188–190). Accordingly, CD38^+^ CLL cells show increased migration, not only in response to chemokines but also in their absence, due to enhanced basal cell spreading and migration (191, 192) (Figure 2). The mechanism involved in the CD38 action was recently shown to involve Ca^2+^-induced activation of the GTPase Rap1, thus providing a novel role for this GTPase in CLL aggressiveness (192). Besides its role in cell migration, CD38 may constitute a marker for activated or/and recently born CLL cell subsets, as CD38 expression is modulated by the microenvironment and has been associated with CLL cell proliferation (193). In line with this, CD38^+^ CLL cells also show enhanced signaling induced by BCR or by anti-IgM/IgD antibody crosslinking, as well as a survival and proliferation advantage, compared to CD38^−^ clones of the same individual (191).

## Selectins

The P-selectin glycoprotein ligand-1 (PSGL-1) is highly expressed on the surface of MM cells (194, 195). *In vitro* and *in vivo* studies have shown that PSGL-1 contributes to MM cell interaction with the BM microvasculature (195), by facilitating the rolling of these neoplasic cells on P-selectin on the endothelium (55). The important roles of the selectins in the initial steps of MM cell homing to the BM were further supported by the demonstration that sialyltransferase ST3Gal-6, an enzyme critical for the generation of E-selectin ligands, is involved in MM cell homing to the BM (196). Moreover, using intravital microscopy, we have demonstrated that antibodies to P- and E-selectin inhibit the rolling and subsequent firm arrest of MM and CLL cells to the BM microvasculature (55). Therefore, both P- and E-selectin seem to control the initial steps of MM and CLL cell homing (Figures 1, 2). With respect to acute leukemias, it has been reported that T-ALL cells adhere to IL-1β-stimulated HUVEC in an E-selectin-dependent manner (197) (Figure 3). In contrast to normal leukocytes and MM cells, where PSGL-1 is the major ligand of P- and E-selectin, B-ALL cells express low levels of PSGL-1 and their rolling and adhesion is mainly mediated through CD43. Of note, CD43 downregulation impairs tissue engraftment in a B-ALL xenograft mouse model (198).

*In vivo* studies of the initial interactions with high endothelial venules have demonstrated a crucial role for L-selectin (CD62L) in CLL cell homing to LNs (199) (Figure 2). This work revealed a higher rolling fraction of cells with high L-selectin expression albeit the rolling velocity was decreased, a fact that may facilitate CLL cell retention in LNs. In line with this, the PI3-Kδ inhibitor idelalisib diminished L-selectin expression, increased rolling velocity and reduced CLL cell entry into LNs (199). BCR engagement was also shown to downregulate CD62L as well as CXCR4, and to favor cell arrest in LNs, in this case by inducing an adhesive phenotype (200, 201).

## Functional Molecular Complexes: the CLL Cell Invadosome

Functional and/or physical associations among molecules involved in CLL cell trafficking have been well-documented. We have shown that ligand engagement of the α4β1 integrin upregulates MMP-9 expression and induce its localization in podosomes, where MMP-9 degrades extracellular matrix and facilitates cell migration (175). Likewise, the CXCL12/CXCR4 or CCL21/CCR7 axes also upregulated MMP-9, involving different pathways than those upregulating α4β1, and increased CLL cell migration (164, 175). Additionally, MMP-9 binds to a CLL cell-specific functional complex formed by α4β1 and CD44v, and this association is important for CLL cell migration and survival (48, 78). Similar to α4β1 and to the chemokine receptors CXCR4 and CCR7, CD38 signaling was also shown to upregulate MMP-9 expression and function in CLL (202). CD38 also synergized with the chemotactic function of CXCL12 and enhanced the adhesive and signaling activity of α4β1 in CLL (202, 203). Further analyses by these same authors demonstrated a physical association of CD38 with CXCR4 and α4β1 at the CLL cell membrane, which provides a possible explanation for the observed functional cross-talk between these proteins (202, 203). In agreement with these studies, Buggins et al. (204) reported the presence of the MMP-9/α4β1/CD44/CD38 macromolecular complex in CLL cases with poor prognosis. CLL cell trafficking between PB and lymphoid tissues therefore involves multiple molecules, which seem to interact physically and/or functionally to form macromolecular complexes. While these complexes may represent the CLL invadosome, as previously suggested (205) (Figure 2), certain issues regarding their dynamic formation, regulation by the microenvironment, turnover, differential composition, etc., still need to be resolved. The fact that the MMP-9/α4β1/CD44/CD38 complex was mainly observed in poor prognosis cases reinforces the importance of molecules regulating migration as contributors to CLL progression.

## Trafficking molecules as Therapeutic Targets in Hematologic Tumors

Because MM, CLL, and ALL progression involves critical infiltration and retention of malignant cells in lymphoid tissues, targeting the molecules that control the cancer cell traffic appears as an efficient therapeutic approach to treat these diseases. Strategies aimed to target α4β1 integrin have proven difficult due to the essential physiological function of this integrin in lymphocyte development and traffic. A recombinant humanized anti-α4 monoclonal antibody (natalizumab) was shown to block stroma-dependent MM cell proliferation, to inhibit *in vivo* tumor growth, and to chemosensitize MM cells to BTZ (206). However, treatment with natalizumab entails several side complications, such as the activation of the John Cunningham (JC) virus and associated progressive multifocal leukoencephalopathy (207), as well as the unwanted egress of normal hematopoietic stem cells linked to inhibition of α4β1 function (208). For these reasons, treatment of hematologic malignancies with natalizumab has been discontinued.

Targeting the signaling pathways that control the α4β1 integrin may be a more suitable therapeutic approach. An important regulator of α4β1 is the BCR, and several studies have demonstrated that inhibiting the BCR-target BTK with ibrutinib abolishes the CLL adhesive and migratory function of α4β1 in tissues, a fact that correlates with the observed ibrutinib-induced lymphocytosis (209, 210) (Figure 2). A more recent report has shown that BCR activates α4β1 integrin and that ibrutinib only partially reduces this activation, since ibrutinib-treated cells were still able to activate α4β1 in response to anti-IgM stimuli (211). These authors suggested that CLL cells with high levels of α4β1 are more likely to be retained in tissues, and that analysis of α4β1 expression may help identify patients which would benefit from ibrutinib therapy

In ALL, the small molecule inhibitor of the α4 subunit TBC3486 blocks ALL cell adhesion, reduces α4 expression, sensitizes B-ALL cells for death *in vitro* and extends survival time in a B-ALL xenograft model (212). In addition, ATL1102, an α4 antisense oligonucleotide developed to treat multiple sclerosis, downregulates the expression of α4 and β1 subunits in the B-ALL Kasumi-2 cell line *in vitro* (213). Unfortunately, this antisense oligonucleotide fails to downregulate α4 expression and to improve survival in a mouse model of B-ALL (213), indicating that antisense drugs must be improved for clinical application in this malignancy. Interestingly, as there is a functional link between α4β1 and the histone methyltransferase G9a, which regulates migration of Jurkat (T-ALL) cells (214), the G9a inhibitor CM-272 has been reported to block cell proliferation and infiltration in an *in vivo* xenograft model (215).

Similar to the high-affinity conformations of the integrins α4β1 and LFA-1, the α4β7 integrin can also display activated conformations that can be recognized by specific antibodies (216). α4β7 has been recently the subject of therapeutic studies in MM using α4β7-based chimeric antigen receptor (CAR) T cells (91). An epitope in the N-terminal region of the β7 subunit (MMG49) is accessible in the α4β7 active conformation but inaccessible in the resting integrin conformer. The study showed that T cells transduced with MMG49-derived CAR exerted anti-MM effects without damaging normal hematopoietic cells, indicating that MMG49 CAR T cell therapy might be promising for MM treatment (91).

The CXCR4-CXCL12 axis has been also the subject of therapeutic studies in MM, CLL and ALL. Thus, *in vivo* CXCL12 inhibition with NOX-A12 led to a tumor microenvironment less receptive for MM cells, causing reduced MM cell growth and disease progression (115) (Figure 1). Furthermore, NOX-A12 increases the anti-MM capability of combined BTZ and dexamethasone (217). In CLL, treatment with NOX-A12 led to inhibition of CLL cell chemotaxis and stroma-mediated drug resistance (Figure 2) (218).

In addition to promoting CD34^+^ mobilization in MM treatments (219), plerixafor augments the sensitivity of MM cells to multiple therapeutic agents (120). Several anti-CXCR4 monoclonal antibodies are being tested in different clinical trials for MM, including ulocuplumab (BMS-936564*/*MDX1338) and hz515H7 (220, 221) (Figure 1), supporting the relevance of targeting MM cell trafficking molecules to inhibit disease progression. Besides ulocuplumab and plerixafor, other monoclonal antibodies or small molecule antagonists to CXCR4, such as BL-8040 and PF-06747143 have been shown to inhibit the CXCL12/CXCR4-mediated migration of CLL cells (222) (Figure 2). Since CXCR4 is also an important survival factor in these cells, some of these antagonists also induce cell apoptosis. Thus, plerixafor and NOX-A12, in combination with rituximab, lenalidomide or bendamustine, are in the initial phases of clinical trials for CLL (222). In the case of ALL, the CXCR4 antagonist POL5551 has been proposed as a possible therapeutic agent in high-risk B- and T-ALL patients (223).

Other chemokines receptors, such as CCR7, CCR9, or CXCR3, have also been considered potential therapeutic targets in CLL and ALL, and the focus of several investigations. Thus, anti-CCR7 mAbs may have potential use to prevent CLL cell traffic to LNs, although they are still in preclinical phases of study (162). In addition, it has been recently described that immunotherapy based on antibodies against CCR9 has anti-tumor potential, without affecting the chemotactic response of T-ALL cells to CCL25 (224). Furthermore, treatment with AMG487, a specific CXCR3 inhibitor, impairs B-ALL infiltration into BM, spleen and brain in *in vivo* models (126).

As mentioned above, CD44 represents an important cell surface molecule for blood cancer cell trafficking. In MM, CD44 has been the focus of CAR T cell studies. T cells targeted to CD44v6 using a CAR construction elicited a potent anti-tumor effect against primary MM and acute myeloid leukemia, while sparing normal hematopoietic stem cells and CD44v6-expressing keratinocytes (225). On the other hand, the humanized anti-CD44 mAb RG7356 is in phase 1 for acute myeloid leukemia and has also shown preclinical activity in CLL (162, 226). Two antibodies against CD38, daratumumab (FDA-approved) and isatuximab (SAR650984; clinical trial not yet completed), have been developed (10, 227), and shown to have anti-MM activity, especially when combined with lenalidomide (228). Likewise, isatuximab is in phase 1/2 clinical trial for CD38^+^ hematologic malignancies, including CLL (227). Additionally, daratumumab has shown preclinical anti-tumor activity in a CLL mouse model, as it eliminates cells from infiltrated organs and inhibits CLL cell homing to spleen (162, 227, 229).

CD19 and the SLAMF7/CS1 receptors are also the subject of therapeutic studies aimed at hematologic malignancies. For instance, CAR T cell therapy directed to CD19^+^ cells is undergoing clinical trials in CLL with promising results (230, 231). Combination of this therapy with approaches addressed to inhibit cell migration may constitute an efficient treatment for CLL. Moreover, CD19-targeted CAR T-cell therapeutics for B-ALL are being developed (232). SLAMF7/CS1 is a putative adhesion molecule mediating MM cell attachment to BM stromal cells (233). A humanized anti-CS1 antibody, elotuzumab (HuLuc63), exerted significant *in vivo* anti-MM activity *via* NK-mediated antibody-dependent cellular citotoxicity (234). In a recent phase 3 study, it was reported that the combination of elotuzumab, lenalidomide, and dexamethasone led to significant reduction in the risk of disease progression or death (235). Furthermore, CAR-engineered NK cells specific for CS1 exhibited significant anti-MM activity both *in vitro* and *in vivo* (236). Collectively, these data indicate that agents targeting adhesion and migration receptors represent promising therapeutic protocols to hamper the progression of blood cancers, including MM, CLL, and ALL.

## Conclusion

Tumor cell migration is a critical process that contributes to the development and progression of hematologic malignancies. Homing and retention of neoplastic cells in tissues involves multiple adhesive and migratory mechanisms and favors survival and chemoresistance of the malignant cells. We have focused this review on the molecular components that regulate malignant cell traffic in three common hematologic tumors, namely MM, CLL, and ALL. These components include integrins, chemokines and chemokine receptors, selectins, metalloproteinases, and other molecules such as CD44 and CD38. These molecules may act in concert at different steps of the trafficking process. Numerous *in vivo* and *in vitro* studies have established the crucial role of the α4β1 integrin and the CXCR4 chemokine receptor in controlling the migration and retention of MM, CLL, and ALL cells in tissues. The role of other molecules (CD44, CD38, MMP-9, selectins) differs among the three malignancies, but they are also important for interactions with the tissue microenvironment. Indeed, most of the migration regulatory molecules also provide survival signaling upon binding to their ligands, thus contributing to progression of the disease. Due to this dual effect, targeting the molecules involved in malignant cell traffic or the signaling pathways that regulate their functions may constitute an efficient therapeutic approach for these diseases. Initial pre-clinical and/or clinical trials with inhibitors for some of these molecules are promising, although the fact that normal lymphocytes use similar migratory mechanisms adds serious difficulties to these approaches. Clearly, more specific targets or signaling pathways need to be identified and tested. Additionally, the development of improved monoclonal antibodies targeting molecules involved in the trafficking of MM, CLL, and ALL cells, such as CXCR4, CD44, SLAMF7/CS1, and CCR9 might open new opportunities for clinical treatments against these hematologic malignancies. Finally, further work on CAR T cell technology, as well as combined therapy using immune check-point inhibitors and agents targeting neoplastic cell trafficking will provide important tools to restrict the progression of these diseases. Additional analyses of the mechanisms involved in the recruitment of neoplastic cells from the bloodstream into different organs using imaging techniques could provide new insights on the migration and tissue-induced survival of malignant cells.

## Author Contributions

JR-M, AG-P, and JT contributed to the preparation of the review article. All three authors reviewed and approved the final version of the manuscript.

### Conflict of Interest Statement

The authors declare that the research was conducted in the absence of any commercial or financial relationships that could be construed as a potential conflict of interest.
